# The Shannon Entropy Trend of a Fish System Estimated by a Machine Vision Approach Seems to Reflect the Molar Se:Hg Ratio of Its Feed

**DOI:** 10.3390/e20020090

**Published:** 2018-01-29

**Authors:** Harkaitz Eguiraun, Oskar Casquero, Iciar Martinez

**Affiliations:** 1 Department of Graphic Design and Engineering Projects, Faculty of Engineering in Bilbao, University of the Basque Country UPV/EHU, E-48013 Bilbao, Spain; 2 Research Center for Experimental Marine Biology and Biotechnology-Plentziako Itsas Estazioa (PiE), University of the Basque Country UPV/EHU, E-48620 Plentzia, Spain; 3 Department of Systems Engineering and Automatic Control, Faculty of Engineering in Bilbao, University of the Basque Country UPV/EHU, E-48013 Bilbao, Spain; 4 IKERBASQUE Basque Foundation for Science, E-48013 Bilbao, Spain; 5 Norwegian College of Fishery Science, Faculty of Biosciences, Fisheries and Economics, University of Tromsø-The Arctic University of Norway, N-9019 Tromsø, Norway

**Keywords:** entropy, seabass, mercury, selenium, fish welfare, machine vision, contaminant detection, biological warning systems, system perturbation, environmental monitoring

## Abstract

The present study investigates the suitability of a machine vision-based method to detect deviations in the Shannon entropy (SE) of a European seabass (*Dicentrarchus labrax*) biological system fed with different selenium:mercury (Se:Hg) molar ratios. Four groups of fish were fed during 14 days with commercial feed (control) and with the same feed spiked with 0.5, 5 and 10 mg of MeHg per kg, giving Se:Hg molar ratios of 29.5 (control-C_1_); 6.6, 0.8 and 0.4 (C_2_, C_3_ and C_4_). The basal SE of C_1_ and C_2_ (Se:Hg > 1) tended to increase during the experimental period, while that of C_3_ and C_4_ (Se:Hg < 1) tended to decrease. In addition, the differences in the SE of the four systems in response to a stochastic event minus that of the respective basal states were less pronounced in the systems fed with Se:Hg molar ratios lower than one (C_3_ and C_4_). These results indicate that the SE may be a suitable indicator for the prediction of seafood safety and fish health (i.e., the Se:Hg molar ratio and not the Hg concentration alone) prior to the displaying of pathological symptoms. We hope that this work can serve as a first step for further investigations to confirm and validate the present results prior to their potential implementation in practical settings.

## 1. Introduction

The forecasted growth for the world human population estimates that there will be somewhere between 9.6 and 12.3 billion people in the year 2100 [1]. As a consequence, one of the main global concerns is to improve and optimize the food production systems, with particular emphasis on seafood [2,3,4], expected to be a main provider of key nutrients such as high quality proteins, omega-3 fatty acids, trace elements and vitamins [4,5].

Unfortunately, and as a result of human activities, the aquatic environment is contaminated with a variety of pollutants, including mercury (Hg), whose organic form (methylmercury, MeHg) is currently considered as one of the regulated contaminants of concern [6] albeit only in seafood [7]. The acceptable maximum level of Hg in most fish products is 0.5 mg/kg wet weight, except for long lived species placed high in the trophic chain for which a level is set at 1.0 mg/kg wet weight.

It is not possible to control the levels of MeHg in the tissues of captured fisheries, but given that the contaminant is accumulated in the food chain, a strict control of the water quality and the feeds’ composition should minimize or eliminate the risk of high MeHg levels in farmed fish. However, this introduces a new challenge, since nowadays it is not possible to completely substitute some marine feed ingredients (in particular those from fatty fish) by others of terrestrial origin without seriously compromising the health and welfare of the fish as well as its nutritional value [8]. Therefore, the maximum level of Hg in feeds destined for fish farming has been set to 0.227 mg Hg per kg dry feed [9]. Interestingly, it has been proposed that the toxicity of Hg is exerted through its ability to interact with selenium (Se) [10], which in turn plays a critical role to maintain the cellular redox potential. Thus, a molar excess of Hg over Se will mean that the cell does not have enough Se to maintain its redox potential and pathological alterations characteristic of Hg poisoning will take place. If, on the other hand, there is Se in excess, then the negative effect of Hg will be neutralized and there will remain enough Se to allow the cells to perform satisfactorily [10,11]. Unfortunately, it is not yet common to refer to the Se:Hg molar ratio when evaluating seafood safety.

Novel, on-line, non-destructive, efficient monitoring systems will also be in demand to ensure the safety and welfare of farmed fish. Targeting this purpose, our research group has proposed the use of fish of the same species being cultivated as a Biological Warning System (BWS) in order to detect the introduction of undesirable contaminants in their environment and/or feed during production [12]. There are several reasons to propose the use of BWS: one is that the organisms themselves will act as integrators of all the substances they are exposed to (i.e., both listed and monitored and new and unexpected substances), another is that contaminants usually occur in mixtures and that the effect of the mixture is not necessarily the sum of the effects of each component alone [10].

Our previous works, based on the principle of a BWS, indicated that the seabass system’s SE was sensible to the presence of MeHg in the environmental water [13] and that the trend of the SE-values over time gave information of relevance to assess the system’s recovery from a temporary contamination [14]. However, the most common route of entrance of MeHg in the fish tissues is through the feed. Therefore, the present work was designed to (i) quantify the response of *D. labrax* systems to feeds containing four different concentrations of MeHg during 14 days and (ii) to evaluate the suitability of the systems’ (ii-a) basal SE, (ii-b) SE of the response to a stochastic event and (ii-c) evolution of these two SE values over time, to verify the presence of MeHg in the system.

## 2. Materials and Methods

The experimental procedure was approved by The Ethical Committee for Animal Welfare No. CEBA/285/2013MG.

### 2.1. Experimental Cases

Four European seabass (*Dicentrarchus labrax*) experimental cases were monitored every second day during 14 days. Each case consisted of 7 fish and they were fed: the control group (C_1_) standard commercial pellets, and the exposed groups (C_2_, C_3_ and C_4_) feed spiked with 0.5, 5 and 10 mg MeHg/kg respectively. In order to minimize the stress to the fish, the video recording was performed every second day, i.e., on the 2nd, 4th, 6th, 8th, 10th, 12th and 14th day. The visual and environmental conditions in each of the four tanks, as well as the biomass, were maintained as similar as possible (Table 1). Prior to the beginning of the exposure, the fish were acclimated for 3 days to the tanks. No mortality or abnormal behavior was observed during the experimental period.

### 2.2. Experimental Set Up and Spiking of the Feed

The experimental set up has been described in [13]. In short, the fish were placed in 4 tanks (100 cm × 100 cm × 90 cm) filled up to 80.5 cm of height with 810 L of aerated circulating seawater. Each tank was under direct artificial light (2 × 58 W and 5200 lm) to avoid shadows, with a 12 h /12 h dark/light photoperiod. The fish were fed INICIO Plus from BioMar (56% crude protein, 18% crude fat) once a day following the manufacturer’s specifications for their size, weight and water temperature. The MeHg doses were selected to reflect realistic contents of Hg in fish (for example 4.54 ppm Hg have been reported in sandbar shark (*Carcharhinus plumbeus*) [15]).

The contaminated feeds were prepared as follows: methylmercury(II) chloride (CH_3_HgCl; Sigma-Aldrich, product number 442534, Mw = 251.08) was first dissolved in DMSO to a concentration of 250 μg MeHg/μL and then diluted with 99% ethanol to 125 μg MeHg/μL. This solution was further diluted with 99% ethanol to produce 10 mL of three solutions: solution A (0.25 μg MeHg/μL), solution B (0.125 μg MeHg/μL) and solution C (0.0125 μg MeHg/μL). Three batches of contaminated feed were prepared by mixing 175 g of feed with 7 mL of solution A, solution B or solution C as follows: the trays were placed inside a hood containing 175 g of feed each, then 7 mL of the each MeHg solution were pipetted to its corresponding tray and the pellets and the MeHg solution were carefully mixed to ensure an even blending of the MeHg containing ethanol solution and the pellets. The trays were covered and left for 3 days inside the hood. Twice a day, each tray was uncovered, its contents mixed and covered again. At the end of the third day, the pellets in the trays appeared dry and we considered that the MeHg had been absorbed by the pellets. This produced three 175 g batches of contaminated feed containing, theoretically 10, 5 and 0.5 μg MeHg/g feed respectively.

Relevant seawater parameters were measured daily prior to feeding. The values, considered optimal, are shown in Table 2. O_2_ saturation was measured with a JBL O_2_ kit, salinity with a HANNA HI98192 meter, NH_3_ with a Sera NH_4_-NH_3_ kit, pH with a Sera pH Kit and temperature with a mercury thermometer. Water flow was estimated based on the intake-water pump load. The water was circulating continuously and it was halted only during the recording periods to allow for good quality video images.

### 2.3. Video Recording Procedure

**Figure 1:**
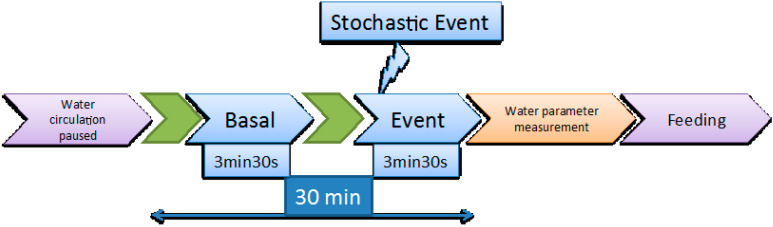
Schematic representation of the video recording procedure.

Video recording was performed every second day as described in [13]. As mentioned above, water circulation and air bubbling were halted immediately before the initiation of the 30 min video recording window. During those 30 min, two video clips of 3.5 min, each corresponding respectively to the basal state and to the response of the fish to a stochastic event, which was a sudden hit in the tank that took place in the 30th s of the recording, were analysed. The procedure is illustrated in Figure 1.

Data acquisition was done by video camera recording using the same experimental setup described in Eguiraun et al. [13,14]. Summarizing, a GoProHero3 camera with underwater housing was used inside each tank. The raw data were recorded with a 1080 p high definition format, 24 frames per second (fps) and 16:9 video size and the videos were locally stored in SanDisk 32 Gb UltraMicroSDHCTM (Class 10) secure cards in each camera.

### 2.4. Video Data Processing, Trajectory Estimationg and Shannon Entropy (SE) of the Trajectories

**Figure 2:**
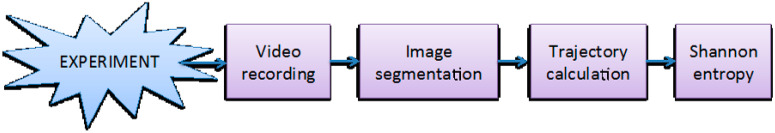
Schematic representation of the data acquisition and processing workflow. For further information see publications [13,14].

The procedure is explained in detail in publications [13,14] and summarized in Figure 2. In short, once the two video clips (1 basal and 1 response per tank and per day) were located in the 30 min recording, they were transformed into a 640 pixel × 480 pixel format image sequences at 24 fps using the iMovie commercial software. Subsequent image and feature extraction were carried out with MATLAB R2014a (MathWorks Inc., Natick, MA, USA) running on a MacBookPro 2.6 GHz Intel Core i7 laptop with a SSD storage disk and 16 Gb of RAM.

The trajectory of the fish cluster’s centroid was initially built by computing the elements centre’s in every single frame, but this led to a very noisy signal. The noise of the signal was reduced by calculating the cluster’s centroid applying the K-means algorithm to the number of elements in each frame using the centres of the elements in the first frame as input coordinates. The trajectories in X and Y were analysed in the same format they were obtained, although they have different scale dimensions. X trajectories have dimension from 0 to 640 and Y trajectories have dimension from 0 to 480 due to the 640 × 480 pixel image size. The results indicated that analysing the raw trajectories leads to satisfactory results and differences were not found between the results obtained analysing the raw and the normalized trajectories. However, and with the purpose of building a more robust algorithm for future applications, the X and Y trajectories presented in the current work were normalized using the Z-score technique.

Finally, the entire image sequences (corresponding to the two 3.5 min video clips) for both the basal and the response to the event were analyzed computing the SE entropy [16] of the trajectory signals of the clusters’ centroid for both axis, X and Y.

SE has found many applications including the evaluation of chaoticness of dynamic systems of arbitrary geometrical shapes [17], redundancy in the English language [16,18] and the complexity of fish trajectories [13,14,19]. In the present work, the SE is used with the sole purpose of characterizing the trajectory signals of each experimental case and performing comparisons among them. We do not have a biological interpretation of what the SE really means in our particular case; for example, whether the fish would move more or less, higher or lower in the water column, clustering together or not, aspects which become complicated by the technical issues already described in our previous work [13] including those related to the 2D analysis of a 3D phenomenon and image segmentation. In any case, we do not intend to use the SE in order to characterize the behavior of the fish or their spatial distribution, which are different issues and would require a different methodological approach.

The SE was formulated by Shannon [16,18] and it is calculated by the equation:

$$H\left(X\right)=-\underset{x_{i}\in \Theta }{\sum }p\left(x_{i}\right)\log\;p\left(x_{i}\right)=-E\left[\log p\left(x_{i}\right)\right]$$

where X represents a random variable with a set of values Θ and probability mass function $p\left(x_{i}\right)=P_{r}\left\{X=x_{i}\right\},x_{i}\in \Theta$, and E represents the expectation operator. Note that p log p=0 if p=0.

### 2.5. Statistics

Statistics were computed using R software.

## 3. Results and Discussion

Large individual variation in responses to environmental changes is usually encountered in biological systems (see [20] and references therein). The current system was no exception, and as shown in Table 1, large variations were noticeable in the weight distributions within each case. It is interesting to note that only fish in C_1_ and C_2_ experimented a significant (*p* < 0.05) increase of over 30% of their initial weight, while the increase in weight in C_3_ and C_4_ was not significant and lower than 14%. This is understandable since organisms subject to stresses have to use a significant amount of their energy to keep their homeostasis and this usually reflects on impaired growth [21,22].

Large variations were also registered in the measured SE values (Table 3), including among measurements performed on the 2nd day, when differences due to the MeHg treatments should have been unnoticeable. Therefore, in order to make comparisons easier, the SE values of the four cases were normalized with respect to the earliest values registered which were estimated on the 2nd day and considered to be 100% of the SE.

**Figure 3:**
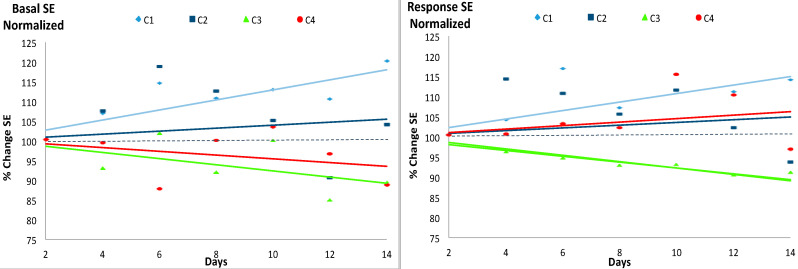
Evolution of the normalized Shannon Entropy (SE) values for the basal state (**left**) and the response to the event (**right**) during the 14 experimental days. The four experimental groups (C_1_, C_2_, C_3_ and C_4_) with 7 individuals each are described in Table 1. Absolute values of the SE are shown in Table 3. The solid lines represent the linear trend of SE from the initial condition at the beginning of the experimental period (normalized SE = 100). For each case, the color of the line is the same as the color of the marker. See Table 4 and Table 5 for further information.

**Figure 4:**
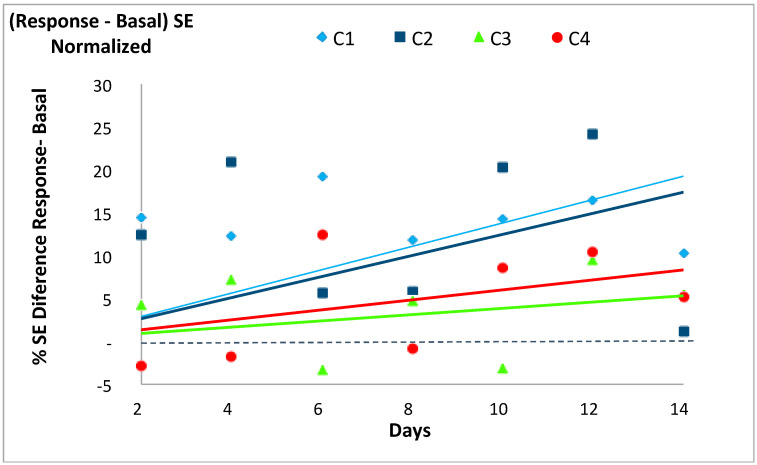
Evolution of the normalized difference between the Shannon Entropy (SE) values for the response to the event minus that of the basal state for each of the four experimental cases (C_1_, C_2_, C_3_ and C_4_) described in Table 1. See Table 6 for further information.

Figure 3 and Figure 4 include the plot of the linear trend of the SE values during the experimental period given the previously proposed potential diagnostic value of such trend [14,19]. The left panel of Figure 3 shows the normalized SE of the basal states. The trends of the SE values divided the 4 cases into two groups: C_1_ and C_2_ on one hand, with a normalized SE value usually higher than 100% and with a tendency to increase and, on the other hand, groups C_3_ and C_4_, with normalized SE values lower than 100% and with a tendency to decrease, although only the regression lines of C_1_ and C_3_ were statistically significant. The parameters of the linear regression lines of the basal state are shown in Table 4.

The normalized SE of the response to the event, shown in the right panel of Figure 3 did not display either a clear relationship with the Hg concentration: in this case however, while the SE values over time of C_1_ were higher than 100% and with a significant tendency to increase and those of C_3_ were lower than 100% and with an also significant tendency to decrease, the trends of C_2_ and C_4_ were similar and tending to increase but not in a significant manner. The parameters of the simple linear regression lines of the response to the event are shown in Table 5.

In accordance with our previous works [14,19] the SE of each system’s basal state was usually lower than its respective SE value in response to the stochastic event, except for two days in C_3_ and three days in C_4_. The differences between the response and the basal SE values for each case tended to increase with time, with a larger increase (i.e., steeper slopes of the trend lines) for C_1_ and C_2_ (slopes 1.38 and 1.25) than for C_3_ and C_4_ (slopes 0.39 and 0.60), as shown in Figure 4 and Table 6. Only the regression line of C_3_ was not statistically significant.

The division of the basal SE evolution of the four systems into two groups, C_1_ and C_2_ on one hand and C_3_ and C_4_ on the other, seems to indicate that the relevant factor to understand such responses as the growth of the fish and the evolution of the SE for each of the four experimental cases is the Se:Hg molar ratio of the feed (C_1_ and C_2_ had a higher than 1 ratio and C_3_ and C_4_ had a lower than 1 ratio) and not the MeHg dose alone. If only the dose of Hg had been the relevant factor, there should have been a dose-related evolution of the SE of the four groups according to the MeHg concentration, which was not the case. This observation is particularly interesting because it means that the SE seems to reflect the effect, and not the concentration, of the MeHg as a contaminant, an effect that is neutralized by dietary Se as indicated by Yamashita et al. [11] and Ralston et al. [23] and therefore supports the recommendation of using the biological system’s SE as a BWS [12]. These results also support Ralston et al.’s [10,23] and Yamashita et al.’s [11] proposition regarding the importance of considering the Se:Hg ratio as the parameter of relevance to determine the toxicity of foods and feeds and not the Hg concentration alone.

## 4. Conclusions and Future Work

We have been able to quantify the response of *D. labrax* systems to feeds containing four different Se:Hg molar ratios during 14 days. Each system’s normalized basal SE gave more consistent results than the normalized SE of the response to a stochastic event and the evolution over time of both the basal SE and of the difference between the response SE minus de basal SE were appropriate to verify the presence of a toxic effect in the system. However, the effect of the toxicant did not seem to be related to the Hg concentration alone: it kept a relationship to the Se:Hg molar ratio of the feeds. Thus, the basal SE of the systems with a molar Se:Hg > 1 (C_1_ and C_2_) tended to increase during experimental period while that of systems with a molar Se:Hg < 1 (C_3_ and C_4_) tended to decrease. In addition, the differences in the SE of the basal states minus the SE in response to the stochastic event were less pronounced in the systems fed with molar Se:Hg < 1. These results were corroborated by the growth of the fish and support the proposition of using the Se:Hg molar ratios as a more reliable criteria to evaluate the risks of MeHg exposure than the levels of Hg alone, as well as the recommendation to use the biological system’s SE as a parameter when implementing a BWS for safety and welfare purposes.

We hope that this work can be used as a first step for future investigations to confirm and validate the present results with more species and settings. Further work should focus on (i) testing the longer-term effect of MeHg contamination as well as the effect of different pollutants (individually and/or in combinations) and doses; (ii) testing alternative data acquisition techniques (sonar, IR) in order to avoid the limitations inherent to video recording [13] and, finally (iii) on analyzing relevant biochemical and physiological parameters on the treated fish in order to understand the interplay between the SE of the biological system and its biological/health status to be able to comprehend the biological meaning of the SE values and tendencies.

## Figures and Tables

**Table 1:** Data on the fish cases showing the weight at the beginning and at the end of the experiment, and the Hg and Se contents in the feeds administered to each case. Each case/tank had 7 fish.

Case	Weight (g) Avg. ± Std.		*p* Value *	mg Hg /kg Feed		Feed Molar Ratio
	Day 0	Day 14		Spiked	Measured **	Se:Hg **
C_1_	141.6 ± 19.2	185.5 ± 40.4	<0.05	0	0.10	29.54
C_2_	138.6 ± 34.9	180.2 ± 10.8	<0.05	0.4	0.47	6.57
C_3_	141.6 ± 18.2	144.2 ± 24.4	n.s.	4	3.9	0.82
C_4_	144.00 ± 22.5	162.9 ± 32.1	n.s.	8	6.7	0.41

* *p* value using the Student’s *t*-test, 1 tail, not paired data and equal or different variance; n.s. = not statistically significant difference; ** The actual Hg and Se concentrations in the feeds were determined by Inductively Coupled Plasma Mass Spectrometry (ICP-MS) in our laboratory by Dr. José Antonio Carrero. The Hg concentration in the commercial feed was within the legal limit, which is 0.227 mg Hg/kg feed [9].

**Table 2:** Seawater parameters during the experimental period.

Parameter	Value
Temperature	16.9–18.5 °C
pH	7.76–7.93
NH_3_	0.0 mg/L
Water flow	0.54 m^3^/h
Salinity	33 g/L
O_2_ Saturation	>80%

**Table 3:** Absolute SE values per experimental day, experimental case (C_1_, C_2_, C_3_ and C_4_) and for the basal state (Basal) and for the response (Resp.) to the introduced event.

Day	C_1_		C_2_		C_3_		C_4_	
	Basal	Resp.	Basal	Resp.	Basal	Resp.	Basal	Resp.
**2nd**	4.37	5.00	5.43	6.10	5.43	5.67	5.07	4.92
**4th**	4.65	5.19	5.82	6.96	5.04	5.43	5.02	4.93
**6th**	4.99	5.83	6.43	6.74	5.52	5.34	4.43	5.06
**8th**	4.82	5.34	6.10	6.42	4.99	5.24	5.05	5.01
**10th**	4.92	5.55	5.69	6.79	5.42	5.25	5.23	5.66
**12th**	4.81	5.53	4.90	6.21	4.59	5.11	4.88	5.41
**14th**	5.23	5.68	5.63	5.70	4.84	5.14	4.49	4.75

**Table 4:** Estimates of the simple linear regressions (y = β_1_ × x + 100) calculated to observe the trend of the SE (dependent variable, y) over time (independent variable, x) from the initial condition at the beginning of the experimental period (SE = 100) for the basal states. A significant regression equation was found in C_1_ (F (1, 6) = 68.65, *p* < 0.001), with an R^2^ of 0.919. A significant regression equation was not found in C_2_. A significant regression equation was found in C_3_ (F (1, 6) = 12.46, *p* < 0.05), with an R^2^ of 0.675. A significant regression equation was not found in C_4_.

	Group	β_1_	Std. Error	t	R^2^
Se:Hg > 1	C_1_	1.294	0.156	8.286 ***	0.919
	C_2_	0.398	0.418	0.952	0.131
Se:Hg < 1	C_3_	–0.760	0.215	–3.529 *	0.675
	C_4_	–0.454	0.243	–1.871	0.368

* *p* < 0.05; *** *p* < 0.001.

**Table 5:** Estimates of the simple linear regressions (y = β_1_ × x + 100) calculated to observe the trend of the SE (dependent variable, y) over time (independent variable, x) from the initial condition at the beginning of the experimental period (SE = 100) for the responses to an event. A significant regression equation was found in C_1_ (F (1, 6) = 33.04, *p* < 0.01), with an R^2^ of 0.846. A significant regression equation was not found in C_2_. A significant regression equation was found in C_3_ (F (1, 6) = 212.1, *p* < 0.001), with an R^2^ of 0.972. A significant regression equation was not found in C_4_.

	Group	β_1_	Std. Error	t	R^2^
Se:Hg > 1	C_1_	1.073	0.187	5.748 **	0.846
	C_2_	0.357	0.359	0.996	0.142
Se:Hg < 1	C_3_	–0.773	0.053	–14.560 ***	0.972
	C_4_	0.454	0.264	1.721	0.330

** *p* < 0.01; *** *p* < 0.001.

**Table 6:** Estimates of the simple linear regressions (y = β_1_ × x) calculated to observe the trend of the normalized difference between the SE values for the response to the event minus that of the basal state (dependent variable, y) over time (independent variable, x) from the initial condition at the beginning of the experimental period (SE of the basal state for each group on the 2nd day = 100%). A significant regression equation was found in C_1_ (F (1, 6) = 16.73, *p* < 0.01), with an R^2^ of 0.736. A significant regression equation was found in C_2_ (F (1, 6) = 6.682, *p* < 0.05), with an R^2^ of 0.527. A significant regression equation was not found in C_3_. A significant regression equation was found in C_4_ (F (1, 6) = 7.092, *p* < 0.05), with an R^2^ of 0.542.

	Group	β_1_	Std. Error	t	R^2^
Se:Hg > 1	C_1_	1.3803	0.3374	4.09 **	0.736
	C_2_	1.2471	0.4824	2.585 *	0.527
Se:Hg < 1	C_3_	0.3860	0.2115	1.825	0.357
	C_4_	0.6008	0.2256	2.663 *	0.542

* *p* < 0.05; ** *p* < 0.01.

## References

[1] Gerland P., Raftery A.E., Sevcíkova H., Li N., Gu D., Spoorenberg T., Alkema L., Fosdick B.K., Chunn J., Lalic N. (2014). World population stabilization unlikely this century. Science.

[2] German Advisory Council on Global Change—WBGU (2013). Governing the Marine Heritage.

[3] Smith M.D., Roheim C.A., Crowder L.B., Halpern B.S., Turnipseed M., Anderson J.L., Asche F., Bourillón L., Guttormsen A.G., Khan A. (2010). Sustainability and global seafood. Science.

[4] Food and Agriculture Organization of the United Nations (FAO) (2016). The State of World Fisheries and Aquaculture 2016. Contributing to Food Security and Nutrition for All.

[5] Organisation for Economic Co-operation and Development (OECD) (2016). The Ocean Economy in 2030.

[6] European Food Safety Authority (2012). Scientific Opinion on the Risk for Public Health Related to the Presence of Mercury and Methylmercury in Food.

[7] European Commission (2006). Commission Regulation (EC) No 1881/2006 of 19 December 2006 Setting Maximum Levels for Certain Contaminants in Foodstuffs (Text with EEA Relevance).

[8] Nasopoulou C., Zabetakis I. (2012). Benefits of fish oil replacement by plant originated oils in compounded fish feeds: A review. LWT Food Sci. Technol..

[9] European Commission (2010). Commision Directive (EC) 2010/6/EU of 9 February 2010 Amending the Annex I to Directive 2002/32/EC of the European Parliament and the Council as Regards Mercury, Free Gossypol, Nitrites and Mowrah, Bassia, Madhuca.

[10] Ralston N.V.C., Blackwell J.L., Raymond L.J. (2007). Importance of molar ratios in selenium-dependent protection against methylmercury toxicity. Biol. Trace Elem. Res..

[11] Yamashita M., Yamashita Y., Suzuki T., Kani Y., Mizusawa N., Imamura S., Takemoto K., Hara T., Hossain M.A., Yabu T. (2013). Selenoneine, a novel selenium-containing compound, mediates detoxification mechanisms against methylmercury accumulation and toxicity in zebrafish embryo. Mar. Biotechnol..

[12] Eguiraun H., Izagirre U., Martinez I. (2015). A paradigm shift in safe seafood production: From contaminant detection to fish monitoring—Application of biological warning systems to aquaculture. Trends Food Sci. Technol..

[13] Eguiraun H., Lopez-de-Ipina K., Martinez I. (2014). Application of entropy and fractal dimension analyses to the pattern recognition of contamined fish responses in aquaculture. Entropy.

[14] Eguiraun H., López-de-ipiña K., Martinez I. (2016). Shannon entropy in a European seabass (*Dicentrarchus labrax*) system during the initial recovery period after a short-term exposure to methylmercury. Entropy.

[15] U.S. Food & Drug Administration Mercury Levels in Commercial Fish and Shellfish (1990–2012). https://www.fda.gov/food/foodborneillnesscontaminants/metals/ucm115644.htm.

[16] Shannon C.E. (1948). A mathematical theory of communication. Bell Syst. Tech. J..

[17] Guariglia E. (2016). Entropy and fractal antennas. Entropy.

[18] Shannon C.E. (1951). Prediction and entropy of printed English. Bell Syst. Tech. J..

[19] Eguiraun H., López-de-Ipiña K., Martinez I. Evolution of Shannon entropy in a fish system (European seabass, *Dicentrarchus labrax*) during exposure to sodium selenite (Na_2_SeO_3_). Proceedings of the 2nd International Electronic Conference on Entropy and its Aplications.

[20] Martinez I., Dreyer B., Agersborg A., Leroux A., Boeuf G. (1995). Effect of T3 and rearing temperature on growth and skeletal myosin heavy chain isoform transition during early development in the salmonid *Savelinus alpinus* (L.). Comp. Biochem. Physiol..

[21] Wendelaar Bonga S.E. (1997). The stress response in fish. Physiol. Rev..

[22] Wendelaar Bonga S.E., Lock R.A.C. (1992). Toxicants and osmoregulation in fish. Neth. J. Zool..

[23] Ralston N.V.C., Raymond L.J. (2010). Dietary selenium’s protective effects against methylmercury toxicity. Toxicology.

